# HCMV-infection in a human arterial organ culture model: effects on cell proliferation and neointimal hyperplasia

**DOI:** 10.1186/1471-2180-7-68

**Published:** 2007-07-20

**Authors:** Rainer Voisard, Tanja Krügers, Barbara Reinhardt, Bianca Vaida, Regine Baur, Tina Herter, Anke Lüske, Dorothea Weckermann, Karl Weingärtner, Wolfgang Rössler, Vinzenz Hombach, Thomas Mertens

**Affiliations:** 1 Department of Internal Medicine II – Cardiology, University of Ulm, Robert-Koch Straße 8, D-89081 Ulm, Germany; 2 Department of Virology, Institute of Microbiology and Immunology, University of Ulm, Robert-Koch Straße 8, D-89081 Ulm, Germany; 3 Department of Urology, Klinikum Augsburg, Stenglin Straße 2, D-86156 Augsburg, Germany; 4 Department of Urology, University of Würzburg, Oberdürrbacher Straße 6, D-97080 Würzburg, Germany; 5 Department of Urology, St. Josef Spital Regensburg, Landshuter Straße 65, D-93053 Regensburg, Germany

## Abstract

**Background:**

The impact of infections with the human cytomegalovirus (HCMV) for the development of atherosclerosis and restenosis is still unclear. Both a clear correlation and no correlation at all have been reported in clinical, mostly serological studies. In our study we employed a human non-injury ex vivo organ culture model to investigate the effect of an in vitro permissive HCMV-infection on cell proliferation and neointimal hyperplasia for a period of 56 days.

**Results:**

During routine-nephrectomies parts of renal arteries from 71 patients were obtained and prepared as human organ cultures. Cell free HCMV infection was performed with the fibroblast adapted HCMV strain AD169, the endotheliotropic strain TB40E, and a clinical isolate (AN 365). After 3, 7, 14, 21, 28, 35, and 56 days in culture staining of HCMV-antigens was carried out and reactive cell proliferation and neointimal thickening were analysed. Successful HCMV-infection was accomplished with all three virus strains studied. During the first 21 days in organ culture no cell proliferation or neointimal hyperplasia was detected. At day 35 and day 56 moderate cell proliferation and neointimal hyperplasia was found both in HCMV-infected segments and mock infected controls. Neointimal hyperplasia in productively HCMV-infected segments was lower than in non infected at day 35 and day 56, but relatively higher after infection with the endotheliotropic TB40E in comparison with the two other strains.

**Conclusion:**

The data do not support the hypothesis that HCMV-infection triggers restenosis via a stimulatory effect on cell proliferation and neointimal hyperplasia in comparison to non infected controls. Interestingly however, even after lytic infection, a virus strain specific difference was observed.

## Background

The role of infection with the human cytomegalovirus (HCMV) for the development of atherosclerosis and restenosis is still unclear. On one hand HCMV DNA was detected in human atherosclerotic vessel walls and in restenotic lesions [1,2] and a possible role of HCMV-infection for restenosis following angioplasty has been discussed [3]. On the other hand conflicting data have been reported from clinical restenosis trials: both a clear correlation [4,5] and no correlation at all have been reported in medium sized clinical studies [6,7]. These studies were based predominantly on HCMV serological data.

A number of critical problems do exist in the field of HCMV research. HCMV replication is restricted to human cells and significant differences exist between HCMV in humans and animal cytomegalovirus models [8]. For that reason data of rat cytomegalovirus infection [9,10] are of limited significance for the human situation. There is no model supporting true HCMV latency in vitro and finally available HCMV strains differ significantly in cell tropism. Especially the widely used laboratory strain AD 169 is highly fibroblast adapted and does not replicate in endothelial cells. In order to obtain suitable data on the effect of HCMV-infection in humans a human model is compulsory. Recently a human ex vivo organ culture model (HOC-model) has been introduced by our group to study the effect of angioplasty on cell proliferation and neointimal hyperplasia [11] for a period of 56 days. In humans and animal models of atherosclerosis, restenosis, and organ transplantation, injury appears to be a critical first step in the acceleration of vascular disease by HCMV. However in order to maintain clear control of the ex vivo model the authors decided to use a human non-injury HCMV model. A corresponding model has been used successfully to study a long lasting productive HCMV infection [12]. In the current study we employed this non-injury human ex vivo model [12] to investigate the effect of HCMV on cell proliferation and neointimal hyperplasia up to 56 days after infection. Nevertheless is has to be kept in mind that it is rather difficult to define a correct and relevant control for a lytically infected organ culture system.

## Results

### Direct visualization of HCMV early antigen

For detailed information of the time course and localization of the infection staining of HCMV early antigen was studied at day 3, 7, 14, 21, 28, 35, and 56 in the plaque-intima, media and adventitia. Productive infection of HOCs was successful with all three strains tested. HCMV early antigen was first detected in the adventitia, thereafter in the plaque-intima and finally in the media.

**Figure 1:**
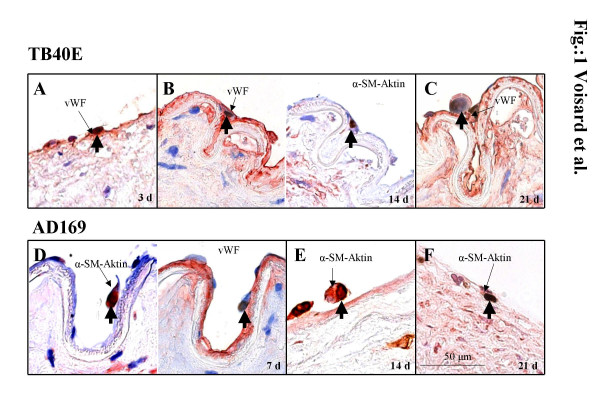
Human organ cultures with double stainings against HCMV-antigens and von Willebrand factor (identification of endothelial cells) or smooth muscle α-actin (identification of smooth muscle cells). The endotheliotropic character of TB40E is demonstrated by the presence of HCMV early antigen after 3, 14, and 21 days (big arrow; A, B, C) within von Willebrand positive cells (small arrow); no smooth muscle α-actin positive cells are found in the area of the TB40E-virus (day 14; B). The tropism of AD169 as fibroblast adapted strain to smooth muscle cells is demonstrated by the presence of HCMV early antigen (big arrow; D, E, F) within α-actin positive cells (small arrow); no positive stainings against von Willebrand factor are found in the area of the AD169-virus (day 7; D).

In extensive kinetic analyses we have shown before [12] that infection with TB40E was slower in comparison to AD169. At day 21 after infection, however, infection was comparable and the area under curve of virus production was almost identical. After infection with TB40E (Fig. 1A–C) endothelial cells became infected more efficiently whereas AD169 (Fig. 1D–F) infected intimal smooth muscle cells [12]. The early productive infection of adventitial cells is probably an artifact of this model in comparison to the in vivo situation.

### Effect of HCMV-infection on cell proliferation

**Figure 2:**
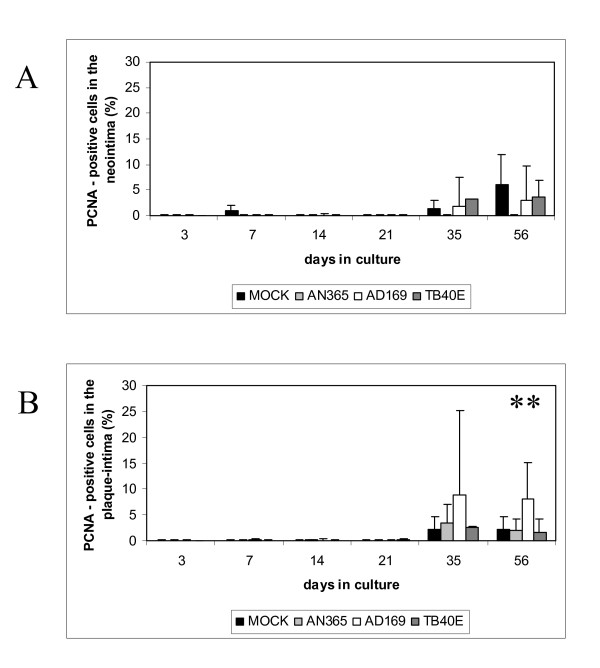
Number of proliferating cells in the neointima (A) and plaque-intima (B) of human organ cultures up to 56 days after infection with HCMV-strains AN365, AD169, and TB40E (** = p = 0.01).

Proliferative response in the neointima, plaque-intima, and media was analyzed at day 3, 7, 14, 21, 28, 35, and 56 (Fig. 2).

In the neointima (Fig. 2A) both in HCMV-infected segments and mock infected controls almost no cell proliferation was found during the first 21 days in culture, except a very modest proliferation at day 7 in mock infected controls (1.1% ± 2.4%). In mock infected controls cell proliferation was increased at day 35 and day 56, 1.5% ± 2.9% (day 35) and 6.0% ± 9.9% (day 56) proliferating cells were detected. In HCMV-infected segments a similar result was found after incubation with HCMV-strains AD169 and TB40E. Proliferating cells were detected in 1.9% ± 5.6% (day 35) and 3.1% ± 6.6% (day 56) after infection with strain AD169 and in 3.2% ± 4.6% (day 35) and 3.7% ± 8.0% (day 56) after infection with strain TB40E. Interestingly, although HCMV infection is cell destructive in vitro at day 35 the number of proliferating cells was even increased in organ cultures infected with TB40E and AD169 in comparison to mock infected controls. Incubation with AN365 did not trigger cell proliferation at day 35 and day 56.

In the plaque-intima a similar result was found (Fig. 2B). Very little cell proliferation was detected both in HCMV-infected segments and mock infected controls during the first 21 days in culture. Cell proliferation in the plaque-intima of mock infected controls was increased at day 35 and day 56, 2.3% ± 4.2% (day 35) and 2.3% ± 6.3% (day 56) proliferating cells were detected. In HCMV-infected segments similar results were detected after incubation with AN365 and TB40E, the number of proliferating cells was 3.5% ± 7.4% and 2.1% ± 3.6% (AN365) and 2.6% ± 6.7% and 1.7% ± 2.8% (TB40E). After incubation with the non endotheliotropic strain AD169 cell proliferation at day 35 and day 56 was increased 3.8 times and 3.5 times (p = 0.01) in comparison to mock infected controls, 8.8% ± 16.3% (day 35) and 8.0% ± 7.1% (day 56) proliferating cells were detected.

In the media of HCMV-infected segments and mock infected controls a similar effect was seen, no matter whether the segments were analyzed at day 3, 7, 14, 21, 28, 35, and 56: no proliferation at all respectively very small rates of proliferation were detected.

### Effect of HCMV-infection on neointimal thickening

**Figure 3:**
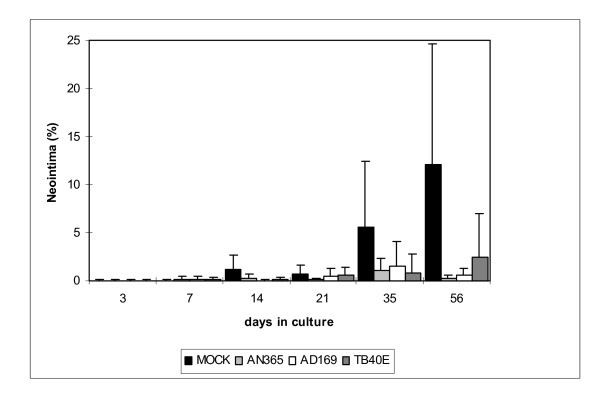
Neointimal thickening in human organ cultures up to 56 days after infection with HCMV-strains AN365, AD169, or TB40E.

Neointimal thickening was analyzed at day 3, 7, 14, 21, 28, 35, and 56 after HCMV infection (Fig. 3). As described earlier by our group [11], preparation procedures triggered neointimal thickening in untreated controls. In uninfected controls no neointima was identified at day 3 and day 7, first very small areas of neointima were found at day 14 and day 21. Neointimal hyperplasia was increased 7.4-fold at day 35 (Fig. 3) and 16.2-fold at day 56 in comparison to neointimal thickening at day 14 (Fig. 3).

After infection with HCMV strains AN365, AD169, and TB40E similarly no or very little neointimal thickening was detected during the first 21 days. At day 35 and day 56 after infection very small areas of neointimal thickening were found. After infection with AN365 neointimal thickening was clearly reduced in comparison to mock infected controls. After infection with strain AD169 and TB40E neointimal thickening was also reduced in comparison to mock infected controls. Interestingly, at day 56 neointimal thickening was pronounced after infection with TB40E in comparison to AD169 and AN365.

### Effect of HCMV-infection on staining against von Willebrand factor and smooth muscle α-actin

Staining of von Willebrand factor in the intima of HOCs was always positive between day 3 and day 56. There was no difference whether HOCs had been infected with HCMV or not.

Positive staining of HOCs against smooth muscle α-actin was always detected between day 3 and day 56, independent of whether plaque-intima or media was analyzed. No staining of smooth muscle α-actin was found in the neointima. No differences were detected between HCMV-infected organ cultures and uninfected controls.

## Discussion

The present study investigates the effect of HCMV-infection on reactive cell proliferation and neointimal thickening in a non-injury human organ culture model for a period of 56 days. In accordance with a recent report of Reinhardt et al. [12] the non injury organ culture model was successfully infected with all three HCMV-strains studied. Therefore this human ex vivo model can be applied for studies of HCMV-infection without the clear restrictions of data produced in animal studies.

The possible molecular mechanisms for the development of vascular disease after HCMV infection is not entirely clear. Streblow et al. [13] reported that the HCMV chemokine receptor US28 may be a potential target in vascular disease. The authors demonstrated that HCMV infections mediates in vitro migration of smooth muscle cells, which was dependent on expression of US28. Reinhardt et al. reported that HCMV infection of human vascular smooth muscle cells leads to enhanced expression of a functionally intact PDGF beta receptor [14] and to reduced expression of extracellular matrix proteins [15]. Recently the group of Stassen et al. (review; [16]) suggested that immune activation following cytomegalovirus infection might be more important than direct viral effects. Despite all these possible mechanisms and alternative pathways the current study investigated the direct effects of HCMV infection in a non injury organ culture model.

HCMV early antigen was first detected in the adventitia, subsequently in the intima and finally in the media. Chen and colleagues [17] reported that HCMV antigen was predominantly found in the inner parts of the vessel wall and were rarely detected in the outer layers. It is obvious that in the human ex vivo organ culture model an atypical path of infection was caused by the cellfree HCMV-infection technique applied. Organ cultures are surrounded by HCMV-infected culture medium and the adventitial side is the largest surface that is presented to the medium.

It has to be noted that it is difficult to generate a relevant control for the infected organ culture system. The formally correct control by an uninfected system neglects the fact that the cell destroying lytic virus infection induced after high titre exogenous in vitro infection causes an additional cell damaging effect. This situation is probably rather different from the in vivo situation with alternating latent states of infection and local recurrences.

In comparison to mock infected controls HCMV-infection did not stimulate cell proliferation and neointimal thickening. At first sight these data do not support the hypotheses that HCMV-infection triggers restenosis, as might have been concluded from experimental data [1-3] and the correlation between HCMV-infection and restenosis in some clinical studies [4,5]. The cell proliferation in mock infected controls was probably caused by the preparation procedures that have to be carried out in all organ culture segments [11]. Although cell proliferation was not increased after HCMV-infection, it remains unclear why some cell proliferation was found after infection with strain AD169 and TB40E but no proliferation at all was detected after infection with strain AN365.

After infection with all three HCMV strains studied neointimal hyperplasia was reduced after 35 and 56 days, reaching statistical significance after day 56. In the case of HCMV strain AN365 the chain of events seems to be understandable, a maximal inhibition of cell proliferation at day 35 caused a significant inhibition of neointimal thickening at day 56. After infection with the HCMV strains AD169 and TB40E however, the significant inhibition of neointimal thickening cannot be explained by an inhibition of cell proliferation. Apart from the contrasting pathways that may have been activated in the organ culture cultures by HCMV-infection [18,19] the high amount of virus applied has to be discussed critically. It is possible that the high amounts of HCMV caused a direct inhibitory effect on intimal hyperplasia. Reinhardt et al. [12] applied high concentrations of HCMV in an identical experimental setting and reported of a productive HCMV-infection even at day 56 after infection, indicating the vitality of the segments.

### Limitations of the study

A major limitation of the model derives from the fact that the segments are stripped off their vasa vasorum. In vivo these vasa vasorum are in charge of supplying the vessel wall with nutrition and oxygen. Only a relative small part of the supply with nutrition and oxygen is delivered by passive diffusion from both the endoluminal and the adventitial surface. In organ culture segments the only supply occurs by passive diffusion of the culture medium, mainly from the adventitial side. These difficulties cause in consequence the unphysiologic path of infection after cellfree HCMV infection. HCMV-infection is starting from the adventitia and moving on to the media, before it's finally approaching the intima. Moreover inflammatory components are missing in the model. Several groups have demonstrated that the acceleration of solid organ rejection in rats requires an active inflammatory component and without it vascular disease does not occur.

During careful surgical extraction all artery segments are treated in the same way according existing standards. All segments are clamped, cautered and cut at the frontal sides. In the laboratory the adventitial side of all segments is further prepared, causing again considerable damage to the vessel. These complex manipulations of all segments cause high standard deviations.

The development of vascular disease, especially atherosclerosis, has been linked to the negative effects of turbulent flow. The model would mimic more closely the in vivo situation if studied in flow conditions. Although first experiences with a human perfused organ culture model have been published by our group [20], HCMV-infection in a perfused human ex vivo model has not yet been reported.

The CMV-status of the donors was not checked in the current manuscript. Reinhardt et al. [12] demonstrated in a similar experimental study design that the level of seropositivity was 60%. However the serostatus of the donor did not influence the kinetic and amount of HCMV-infection in the renal artery segments [12].

Although the authors would like to point out that it is extremely important to model human vascular disease using human tissues and human viruses, due to the limitations of the model a negative result may not truly reflect the natural host:virus complexity.

## Conclusion

The human ex vivo organ culture model was successfully infected with all three HCMV-strains studied. Therefore the model is a suitable basis for further studies of HCMV-infection, without the problems arising from data describing HCMV infection in animal models.

The results of the current study do not support the hypothesis that HCMV-infection might stimulate the restenosis process by activation of cell proliferation and neointimal hyperplasia. However in future studies the issue of an inhibitory effect of high HCMV-levels on otherwise stimulated pathways should be addressed. HCMV-infection in a perfused human organ culture model [20] would more closely mimic the route of infection in vivo.

## Methods

### Preparation and cultivation of human organ cultures

During routine-nephrectomies parts of renal arteries of 71 patients were extracted (male/female: 37/32; mean age: 61.1 years ± 15.4 S.D.). Cylindric segments with a side length of 5 mm were cut from the artery segments. Immediately after preparation, cylinders were transferred as HOCs into sterile HEPES-buffered (15 mmol/L) culture medium (DMEM) without serum supplement.

### Cell free HCMV infection of human organ cultures

For cell free HCMV infection of HOC-segments the laboratory strain AD169, a clinical isolate AN365 and the endotheliotropic strain TB40E were used. Infection of HOC-segments was carried out for 12 h as described by Reinhardt et al. [12] with 5 × 10^5 ^plaque forming units (PFU) of HCMV-strain AD169 and 1 × 10^5 ^PFU of the HCMV strains AN365 and TB40E. After infection with AD169 a reduced amount of HCMV was detected in the culture medium in comparison to the amount of virus after infection with AN365 and TB40E [12]. Therefore an increased amount of AD169 was used in the current study. Infection was carried out in low volume using high titered viral stocks that had been produced in fibroblasts [12]. After cell free infection HOCs were carefully washed in phosphate buffered saline (PBS^+^). As controls HOC-segments without viruses were incubated with the identical sucrosephosphate buffer used for the virus stocks (mock).

### Cultivation and fixation of human organ cultures

HOCs were cultured in a mixture of Waymouth's MB 752/1 and Ham F12 nutrient mixture (1:1 v/v; Bio Whittaker, Verviers, B) supplemented with 15% fetal calf serum (PAA Laboratories, Cölbe, D) at 37°C in 5% CO_2_. The culture medium was exchanged every second or third day. At day 3, 7, 14, 21, 28, 35, and 56 HOCs were washed with PBS^+^, fixed in 4% buffered paraformaldehyde (Merck, Darmstadt, D) and embedded in paraffin. The specimens were cut in series at 4 μm thickness.

### Morphometry of human organ cultures after HCMV-infection

For histological examination deparaffinized sections were stained with an elastica-van Gieson staining (Chroma, Köngen, D). For calculation of the average arterial wall thickening cuts from five (day 3, 7, 14, 21) respectively 10 (day 35, 56) different organ culture segments were analyzed three times for each single experiments with a computerized digital image analyzer (Bioquant TM System 4 R + M Biometrics, Bilancy Consulting, Düsseldorf, D). Taking into account the various lumina of HOC segments, the percentage of neointimal thickening was calculated as: Area of neointima/Area of neointima, plaque-intima and media × 100.

### Immunohistochemistry of human organ cultures

For immunohistological staining paraffin sections were processed using the avidin-biotin immunoperoxidase technique [11].

As primary antibody for the detection of HCMV in HOC-segements HCMV delayed early DNA-binding protein p52: clone CCH2 (DAKOPATTS, Hamburg, D) was used in a concentration of 1 μg/mL. For direct localization of HCMV absolute numbers of HCMV-positive cells was detected in the neointima, media, and adventitia of the sections.

Proliferating cells were identified with PCNA (proliferating cell nuclear antigen, clone PC10, Dakopatts, Hamburg, D). The number of proliferating cells in the media, plaque-intima and neointima was calculated independendly as: Number of PCNA-positive cells/Total cell number × 100. As described cuts from five respectively 10 segments were used in each single experiment at a magnification of ×40.

Smooth muscle cells (SMC) were identified by positive staining with antibodies against smooth muscle α-actin (Renner, Darmstadt, D) at a concentration of 1 μg/mL. Each area of the sections was evaluated at a magnification of ×40 according the criteria: staining positive or staining negative.

Endothelial cells (EC) in HOC sections were identified by positive staining with antibodies against von Willebrand factor (DAKOPATTS).

Double stainings were carried out with antibodies against HCMV early antigen/von Willebrand factor and HCMV early antigen/smooth muscle α-actin.

As secondary antibody a biotinylated horse-anti-mouse antibody (Camon, Wiesbaden, D) was used in PCNA, HCMV-, and smooth muscle α-actin-staining and a biotinylated goat-anti-rabbit antibody (Camon) in von Willebrand staining.

### Statistical analysis

Data are presented as mean ± S.D. The two-tailed *U*-test of Mann and Whitney was used for determining statistical significance of differences. Statistical significance was accepted for *P *< 0.05 [11].

## Authors' contributions

All authors read and approved the final manuscript. RV, BR, BV, RB, VH, and TM designed the study, RV wrote the manuscript. BR, BV, and AL carried out cell free HCMV infection of the renal artery segments. Preparation and cultivation of the segments, identification of the HCMV strains, analysis of cell proliferation and neointimal hyperplasia was done by TK, RB, and TH. DW, KW, and WR supplied renal artery segments and critically discussed the manuscript.
